# Parent-of-Origin Effects on Seed Size Modify Heterosis Responses in *Arabidopsis thaliana*

**DOI:** 10.3389/fpls.2022.835219

**Published:** 2022-03-07

**Authors:** Rosa Castillo-Bravo, Antoine Fort, Ronan Cashell, Galina Brychkova, Peter C. McKeown, Charles Spillane

**Affiliations:** Genetics and Biotechnology Lab, Plant and AgriBiosciences Research Centre, Ryan Institute, National University of Ireland Galway, Galway, Ireland

**Keywords:** Parent-of-origin effects, seed size, hybridity, heterosis, parental genome dosage, ploidy, paternal contribution, *Arabidopsis*

## Abstract

Parent-of-origin effects arise when a phenotype depends on whether it is inherited maternally or paternally. Parent-of-origin effects can exert a strong influence on F1 seed size in flowering plants, an important agronomic and life-history trait that can contribute to biomass heterosis. Here we investigate the natural variation in the relative contributions of the maternal and paternal genomes to F1 seed size across 71 reciprocal pairs of F1 hybrid diploids and the parental effect on F1 seed size heterosis. We demonstrate that the paternally derived genome influences F1 seed size more significantly than previously appreciated. We further demonstrate (by disruption of parental genome dosage balance in F1 triploid seeds) that hybridity acts as an enhancer of genome dosage effects on F1 seed size, beyond that observed from hybridity or genome dosage effects on their own. Our findings indicate that interactions between genetic hybridity and parental genome dosage can enhance heterosis effects in plants, opening new avenues for boosting heterosis breeding in crop plants.

## Introduction

Hybridisation is widely used in crop breeding to harness hybrid vigour effects and for generation of improved phenotypes (Goulet et al., 2017). Improvement of F1 hybrid performance relative to parental lines is known as heterosis and can affect yield, biomass and many other characters (Birchler et al., 2010; McKeown et al., 2013a; Schnable and Springer, 2013; Miyaji and Fujimoto, 2018; Liu et al., 2020). Heterosis breeding schemes underpin higher-yielding F1 hybrid varieties of many crops, including maize, rice, sugar beet, oilseed rape, and sorghum (Cheng et al., 2007; Ma and Yuan, 2015; Goulet et al., 2017; Dimitrijevic and Horn, 2018; McGrath and Panella, 2018; Wolko et al., 2019). Both positive and negative heterosis can be useful, increasing or decreasing a trait value relative to the parents, respectively (Ryder et al., 2014). While positive heterosis is preferable for yield, negative heterosis for growth duration is also useful for earliness in rainfed crops (Kumar et al., 2015).

Seed/grain size is a prime breeding target and a major contributor to seed weight, ultimately influencing yield (Shomura et al., 2008; Kesavan et al., 2013; Makino et al., 2020). Despite the traditional yield gain attributed to seed/grain number, modern crop varieties have demonstrated that seed/grain weight, and therefore seed size, can also be associated with grain yield, driven by artificial selection which can alter the constraints on the trade-off between seed size and number (Griffiths et al., 2015; Golan et al., 2019; Calderini et al., 2021; Martin, 2021). The non-additive mode of inheritance that characterises heterosis prevents the prediction of F1 hybrid phenotypes from performance in the parental generation, which necessitates laborious field testing of hybrids developed from complex breeding designs to identify germplasm pools with good combining ability (Fridman, 2015; Seymour et al., 2016; Vasseur et al., 2019). In this context, the model plant *Arabidopsis thaliana* (hereafter *Arabidopsis*) provides an opportunity for studying parental genotype combinations that generate heterosis effects (Stokes et al., 2007; Schnable and Springer, 2013) due to the number of genotyped accessions (Weigel, 2012). Indeed, seed size heterosis has been reported in some *Arabidopsis* intraspecific hybrids (Alonso-Blanco et al., 1999; Stokes et al., 2007; House et al., 2010; Groszmann et al., 2014; van Hulten et al., 2018; Wang et al., 2020), making *Arabidopsis* a suitable system for improving understanding of the control of seed size heterosis.

Seed size is a life-history trait that is also influenced by parent-of-origin effects, which can occur when the phenotype differs according to whether it is transmitted maternally or paternally (Spillane et al., 2002; Vielle-Calzada et al., 2002; Lawson et al., 2013). Analysis of reciprocal crosses in several species have demonstrated strong maternal effects on F1 seed size, while paternal effects are only rarely reported (de Jong and Scott, 2007; Diggle et al., 2010; House et al., 2010; de Jong et al., 2011; Li and Li, 2015; Singh et al., 2017; Li et al., 2019). The mechanisms underlying maternal effects on seed size include (1) uniparental inheritance of cytoplasmic organelles, usually via the female gametes (although paternal inheritance/leakage of organellar genomes has been reported in certain species), (2) gametophytic effects involving the egg cell and/or central cell, (3) sporophytic effects (mediated by e.g., maternal integuments, the seed coat), and (4) genomic imprinting in the seed endosperm (Grossniklaus et al., 2001; Donohue, 2009; Diggle et al., 2010; Greiner et al., 2015; Armenta-Medina and Gillmor, 2019; Kirkbride et al., 2019).

Paternal effects can also influence seed development in a range of ways, although these are more limited than for the maternal parent. Paternal effects can occur through male gametogenesis or post-fertilisation, due to factors deposited by the sperm cells in the egg and/or central cells, as well as by genomic imprinting (Spillane et al., 2002; Wolff et al., 2015; Pires et al., 2016; Batista and Köhler, 2020). Indeed, the role of sperm-accumulated transcripts (including coding and non-coding RNAs) is apparent in early development in both animals and plants (Gòdia et al., 2018). For example, interruption of the delivery of small RNAs by the sperm causes arrested zygotic divisions in mouse, leading to embryonic lethality (Yuan et al., 2016). In *Arabidopsis*, the sperm-transmitted microRNA, miR159, triggers endosperm nuclear divisions (Zhao et al., 2018), while the sperm-delivered *SHORT SUSPENSOR* (*SSP*) mRNA is necessary for the first zygotic division (Bayer et al., 2009). Epigenetic modifications present in sperm and/or semen can influence offspring development in many taxa, including insects, fish, and nematodes (Macartney et al., 2018) and include inheritance of DNA methylation patterns and chromatin structure, potentially affecting the embryo’s transcriptional programs (Skinner et al., 2015; Klosin et al., 2017; Macartney et al., 2018).

Studies in *Arabidopsis* demonstrate that altering the relative dosages of the parental genomes, typically in inter-ploidy crosses, causes particularly strong parent-of-origin effects on seed endosperm development, resulting in dramatic seed size changes even in the absence of genetic hybridity (Scott et al., 1998; Fort et al., 2016; Lafon-Placette and Köhler, 2016). While doubling the paternal genome dosage in crosses between diploid (2x) and tetraploid (4x) parents can result in prolonged endosperm proliferation and abnormally large seeds which frequently abort, the reciprocal cross between a 4x maternal parent and a 2x paternal parent (generating a maternal genome excess in the F1 seed), causes precocious endosperm cellularisation leading to small F1 seeds (Scott et al., 1998; Fort et al., 2016; Lafon-Placette and Köhler, 2016). We previously demonstrated that paternal excess F1 triploids stably demonstrated heterosis for biomass in a panel of ten accession combinations, in addition to any heterosis seen in crosses between diploids of the same accessions. We could not however associate this with any increase in relative growth rate and concluded that the effect is likely to be due to paternal genomes driving an increase in seed size (Fort et al., 2016).

In this study, we test this hypothesis across *Arabidopsis* genetic diversity by reciprocally crossing 71 accessions to tetraploid tester lines. We quantify the contribution of both the maternal and paternal genomes on F1 seed size and investigate associations with the extent of seed size heterosis. Finally, using reciprocal pairs of maternal and paternal genome excess F1 triploids, we investigate whether parental genome dosage can enhance hybridity-associated parent-of-origin effects on F1 seed size in pairs of equivalent reciprocal F1 hybrid diploids beyond that achieved by polyploidy alone. This analysis allows us to quantify the impact of natural variation on parent-of-origin effects for F1 seed size in crosses between plants of equal or unequal ploidy, and to determine whether there are modifier effects on seed size heterosis arising from the interactions between hybridity and parental genome dosage.

## Materials and Methods

### Plant Growth Conditions and Crossing

*Arabidopsis* seeds were surfaced-sterilised with chlorine gas for 1 h by mixing 30 ml of sodium hypochlorite (10%) and 3 ml of hydrochloric acid (HCl) (37%) in a bell chamber. Sterile seeds were stratified for 3–4 days at 4°C in the dark and germinated directly in 5:1:1 mixture of compost:vermiculite:perlite (National Agrochemical Distributors Ltd., Dublin, Ireland). Plants were grown in a randomised block design under long-day conditions (16 h light, 21 °C /8 h dark, 18 °C) with light intensity 120–150 μE m^–2^ s^–1^ and humidity 70%. Manual crosses were performed on a Leica MZ6 stereo microscope; only flowers on the primary stem were emasculated to reduce possible alterations in seed size due to natural variation between branches. Sepals, petals and stamens were removed using #5 Forceps (Dumont, Montignez, Switzerland) and stigmas allowed to mature for two days before being pollinated.

### Plant Material and Crossing Design

The accessions used in this study and the geolocation of where they were collected (Supplementary Table 1) were provided by Arthur Korte from University Würzburg. The 71 accessions (plus the L*er*-0 tester line) used to generate the reciprocal F1 hybrids are part of an original panel of 400 accessions designed by Arthur Korte which comprised 200 Swedish accessions (Long et al., 2013) and an additional random set of 200 accessions derived from a wide geographic range including Central and Western Europe, Asia, and North America. Tetraploid L*er*-0 seeds (4x L*er*-0) were the kind gift of Brian Dilkes (Purdue University, West Lafayette, IN, United States), generated by colchicine doubling (Blakeslee, 1941). A diploid tester line in L*er*-0 background (2x Ler-0) was used to generate the set of 142 L*er*-0/Accession hybrids (including reciprocals). To minimise any possible environmental effects, three replicates of every cross combination were generated (for a total of 426 manual crosses) and grown in a randomised block design. The seed size value of parental lines was obtained from three biological replicates of self-fertilised plants.

### Seed Size Analysis

Approximately 20 DAP (Days After Pollination) siliques were harvested once they had turned brown before dropping seeds and stored in paper envelopes before seed size analysis. Dried silique material and aborted seeds were removed using forceps, seeds were spread onto an EPSON Perfection V600 Photo Scanner and evenly separated using a painting brush to ensure further single-seed measurements. Images were taken at a resolution of 900 dpi in a black and white format. ImageJ was used to measure seed area (Abramoff et al., 2004), with a range of 0.08–0.3 mm^2^ for diploid and paternal-excess triploid seeds and 0.03–0.2 mm^2^ for maternal-excess triploid seeds to exclude any non-seed material.

### Calculation of Heterosis, Genetic Distance, and Heritability

The mean size (mm^2^) of exclusively plump seeds was used for seed size heterosis calculations following Falconer and Mackay (1996) to evaluate F1 performance over their mid-parent (MP), best-parent (BP), or worst-parent (WP) values (Falconer and Mackay, 1996). Mid-Parent Heterosis (MPH) levels were determined as: %MPH = (F1 – MP)/MP × 100, where the MP value consists of the mean value of both parents. MPH is a relative measure to assess whether F1 hybrids have a greater performance (bigger seed size) than the average performance of both parents. In contrast, Best-Parent Heterosis (BPH) levels evaluate the F1 hybrid value compared only to the best performing parent (BP), determining offspring with increased seed size over the largest parent. BPH levels were calculated as: %BPH = (F1 – BP)/BP × 100. Worst-Parent Heterosis (WPH) was calculated as %WPH = (F1 – WP)/WP × 100 to determine F1 hybrids with a smaller seed size than the worst performing parent (WP). The relative genetic distance was calculated as the proportion of alleles in an accession of a different genotype than that of the tester line L*er*-0, given the assumption of complete homozygosity across all accessions. Calculations were made using a subset of 3,866,657 SNPs acquired from 1001 genomes rather than an absolute distance using all 10,709,949 available SNPs, with a SNP excluded when all 71 accessions as well as L*er*-0 were found to share a genotype at that locus. For each of the cross directions, heritability was calculated by taking the proportion of variance explained by genotype as a fraction of overall variance. Variance were calculated using a random effects model such that:


$$Y_{i\,j}=g_{i}+\varepsilon_{i\,j}$$

where *Y*_*ij*_ is the jth seed of the ith accession cross with L*er*-0, *g*_*i*_ is a factor accounting for the effect of the ith accession and *?_*ij*_* is the error associated with each measurement. The above equation was used to calculate the variance components of the 2x L*er*-0 X 2x Accession cross progenies and the 2x Accession X 2x L*er*-0 cross progenies independent of one another. Heritability in this case is assumed to be broad-sense rather than narrow-sense as the inbred nature of the parental accessions means that all F1 offspring generated by a given cross should be genetically identical.

## Results

### Parent-of-Origin Effects on Size of F1 Hybrid *Arabidopsis* Seeds

**FIGURE 1:**
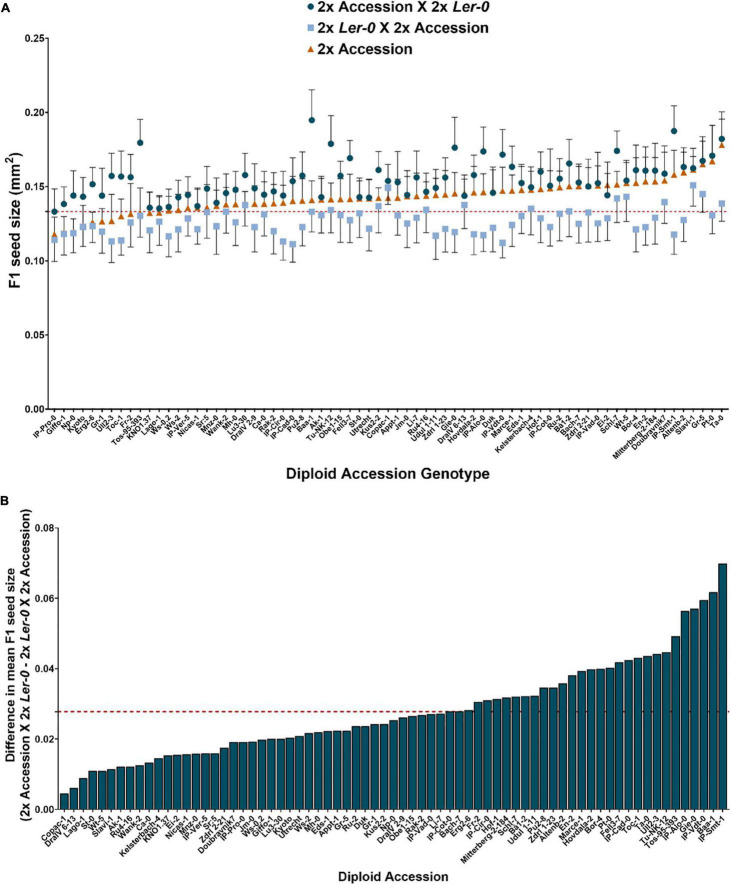
Heterosis effects on F1 seed size observed in reciprocal F1 hybrid diploid seeds depends on parent-of-origin effects. **(A)** F1 hybrid diploid seed size of reciprocal crosses using 2x L*er*-0 as pollen donor (represented by blue circles) or as maternal parent (light blue squares). Seed size of selfed isogenic parental lines is also represented (orange triangles) for comparison with their reciprocal F1 offspring. The horizontal red dashed line corresponds to the mean seed size value of the accession 2x L*er*-0 (0.1332 mm^2^). Error bars represent SD. N ≥ 40 seeds/genotype. **(B)** Natural variation for parent-of-origin effects on seed size across 71 reciprocal F1 hybrid diploids. The red dashed line represents the average difference in mean F1 seed area for the entire set of 71 reciprocal hybrids.

To determine the extent of parent-of-origin effects on F1 seed size heterosis from crosses between different *Arabidopsis* accessions, a panel of 71 genetically different diploid (2x) accessions (Supplementary Table 1) and a 2x tester accession in L*er*-0 background were used as crossing parents to generate reciprocal L*er*-0/Accession F1 hybrids. As a baseline, we first determined the F1 seed size of each parental line when grown under controlled, randomised conditions. As expected, we observed significant natural variation for seed size across the accessions when selfed (Figure 1A), ranging from the small-seeded accessions IP-Pro-0 (0.1179 mm^2^) and the L*er*-0 tester accession (0.1332 mm^2^), to the considerably larger seeds of Pt-0 (0.1672 mm^2^) and Ta-0 (0.1781 mm^2^).

To systematically quantify the extent of parental effects, each cross was performed in both directions (to generate a set of reciprocal F1 hybrid seeds (2x L*er*-0 X 2x Accession vs 2x Accession X 2x L*er*-0). We found that F1 seed size was strongly influenced by parent-of-origin effects, as many pairs of reciprocal crosses displayed large parental effect differences in F1 seed size. Generally, L*er*-0 when used as maternal parent produced the smallest seeds (0.82 times smaller on average than their reciprocal counterparts), whereas L*er*-0 used as pollen donor led to substantially larger F1 seeds (Figure 1A). These results are consistent with previous findings of parent-of-origin effects on seed size in crosses involving L*er*-0 (see section “Discussion”). To quantify the relationship between maternal or paternal genotype and F1 seed size, we performed a correlation analysis (Table 1). A strong correlation (*r* = 0.5285, *p*-value ≤ 0.0001) was found between the seed size of F1 hybrid diploid seeds derived from 2x Accession X 2x L*er*-0 crosses and the seed size of that of the accessions used as maternal parent, confirming that F1 seed size is highly influenced by maternal effects. Notably, we also found a strong positive correlation (*r* = 0.4658, *p*-value ≤ 0.0001) between the seed size of F1 hybrid seeds from 2x L*er*-0 X 2x Accession crosses and the parental seed size of the 2x accessions used as pollen donors, suggesting that F1 hybrid seed size can also be significantly influenced by the paternal genome (Figure 1A). Such “paternal effects” have been reported before from small numbers of inter-accession crosses (House et al., 2010), but have typically been found to be quite small.

**TABLE 1:** Influence of parental seed size on F1 seed size heterosis in hybrid offspring.

	2x Accession X 2x Ler-0	2x Accession
2x *Ler-0* X 2x Accession	*r* = 0.2324 R^2^ = 0.054 *p* value = 0.0512	*r* = 0.4658 R^2^ = 0.217 *p* value ≤ 0.001
2x Accession X 2x *Ler-0*		r = 0.5285 R^2^ = 0.2794 *p* value ≤ 0.001

*Pearson correlation coefficient (r) was used to determine the association between F1 seed size of F1 hybrid diploids and seed size of genetically different accessions used as parental lines. Correlation is significant at the 0.05 level.*

**FIGURE 2:**
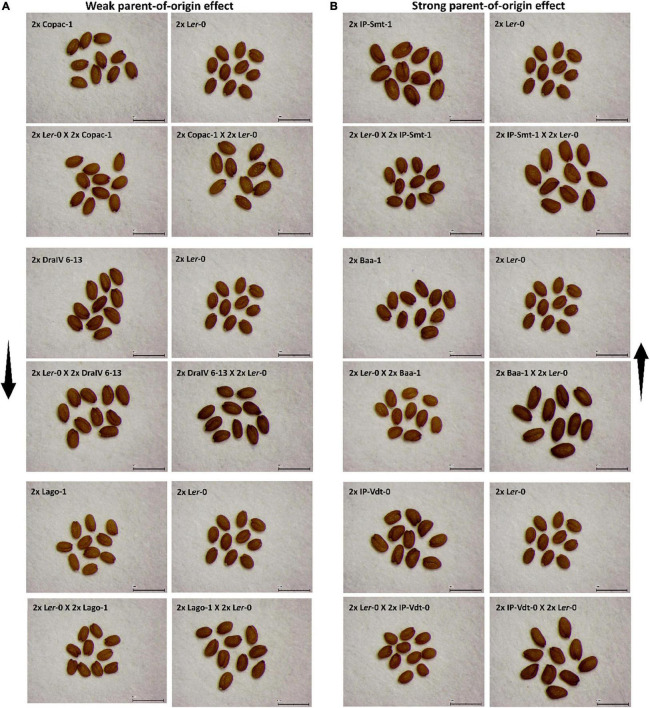
Seed size phenotypes of reciprocal F1 hybrid diploid seeds and their parental lines. **(A)** The three F1 hybrid diploids with the weakest parent-of-origin effects on F1 seed size in this study and the accessions used as parental lines. The black arrow represents the intensity of parent-of-origin effects in ascending order. **(B)** The three F1 hybrid diploids with the strongest parent-of-origin effects in this study and their parental lines. The black arrow represents the intensity of parent-of-origin effects, from lowest to highest. Scale bar = 1 mm.

To quantify the extent of parent-of-origin effects observed in F1 seed size between pairs of reciprocal F1 hybrid diploids, the mean seed size area of F1 seeds from 2x L*er*-0 X 2x Accession crosses was subtracted from the mean F1 seed size of the reciprocal cross 2x Accession X 2x L*er*-0. The positive values obtained (Figure 1B) confirmed the consistently bigger seed size of 2x Accession X 2x L*er*-0 F1 hybrids. Not all genetically different F1 hybrids displayed the same magnitude in seed size differences between pairs of reciprocal F1 hybrid diploids (Figure 1B). For instance, the parental accessions Copac-1, DraIV 6–13, and Lago-1 generated the three F1 hybrids with the weakest parent-of-origin effects (Δ = 0.0045 mm^2^, Δ = 0.0061 mm^2^, and Δ = 0.0089 mm^2^, respectively), while IP-Vdt-0, Baa-1 and IP-Smt-1 were the three parental accessions with the strongest parent-of-origin effects on F1 seed size (Δ = 0.0595 mm^2^, Δ = 0.0617 mm^2^, and Δ = 0.0698 mm^2^, respectively). As shown in Figures 1B and 2, weak parent-of-origin effects led to similar F1 seed size between reciprocal F1 hybrid diploids, whereas strong parent-of-origin effects led to dramatic differences in F1 seed size.

### Extent of Heterosis Effects on F1 Seed Size Strongly Influenced by Parent-of-Origin Effects

**FIGURE 3:**
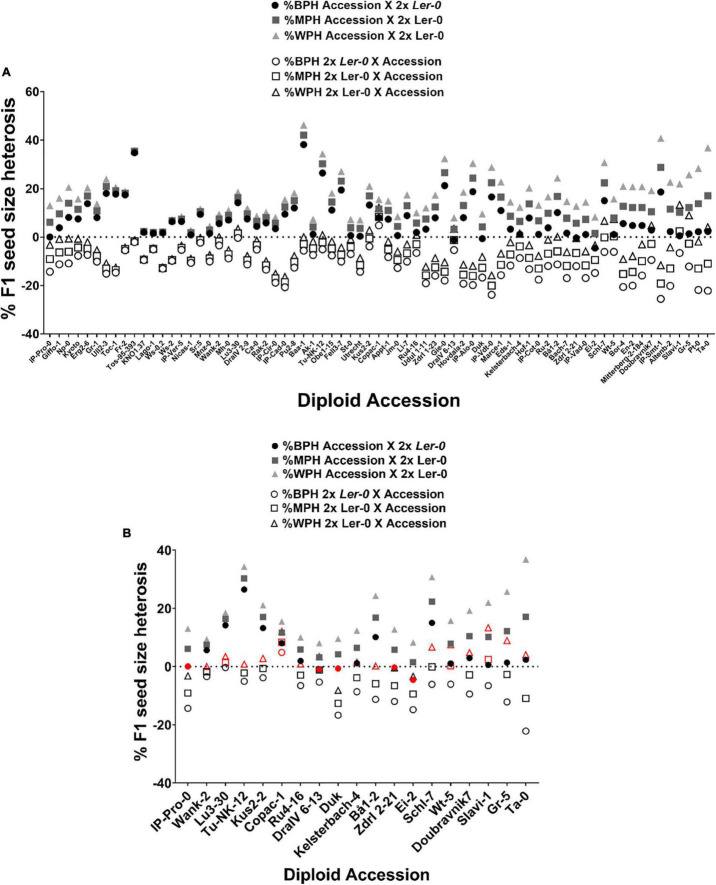
The heterotic effect on F1 diploid seed size depends on parent-of-origin effects. **(A)** Levels of Best-Parent (%BPH), Mid-Parent (%MPH) and Worst-Parent Heterosis (%WPH) across reciprocal F1 hybrid diploid seed size. **(B)** F1 hybrids that do not follow the trend observed among 71 reciprocal L*er*-0/Accession F1 hybrids for %BPH, %MPH or %WPH. Exceptions are indicated in red. The horizontal dotted line represents the critical value 0.

Seed size in *Arabidopsis* inter-accession hybrids frequently demonstrates heterosis when compared to the parental values. To quantify the extent of heterosis on size of F1 seeds and of any impact of the parent-of-origin effects described above, we calculated Best-Parent (%BPH), Mid-Parent (%MPH) and Worst-Parent Heterosis (%WPH) for F1 seed size for all 71 reciprocal L*er*-0/Accession hybrids (Figure 3 and Table 2). Best-Parent Heterosis was found for F1 seed size in all 2x Accession X 2x L*er*-0 cross direction F1 hybrids, except those derived from the parental accessions DraIV-6–13 (BPH = –1.17%), Duk (BPH = –0.68%), Zdrl 2–21 (BPH = –0.4%), Ei-2 (BPH = –4.57%), and IP-Pro-0 (BPH = 0%) (where a negative value for %BPH indicates that the F1 hybrid does not exceed the seed size of the BP value). Notably, IP-Pro-0 when used as maternal parent produced F1 hybrid seeds that were the same size as the BP value (Figure 3B and Supplementary Table 2). All four accessions did, however, display Mid-Parent heterosis, meaning that the F1 hybrids exceeded the seed size of the average value of the two parental lines. The highest levels of BPH were found in Baa-1/L*er*-0 F1 hybrids (following the convention the maternal parent is always mentioned first), whose F1 seeds were nearly 40% larger than the BP value, whereas the lowest levels of BPH were found in the Utrecht/L*er*-0 F1 hybrids, with seeds only 0.4% larger than the BP value.

**TABLE 2:** Hybridity-associated parent-of-origin effects on seed size in F1 hybrid triploids compared with their diploid counterparts.

Accession	2xA X 2xL	2xL X 2xA	POE 2x	2xA X 4xL	4xL X 2xA	POE 3x	2xL X 4xL	4xL X 2xL	genome dosage POE 3x	hybridity-specific POE 3x	3x *vs* 2x
Copac-1	0.1538	0.1493	0.0045	0.1973	0.0911	0.1062	0.1682	0.0914	0.0768	0.0294	6.53333
DraIV 6-13	0.1438	0.1377	0.0061	0.1994	0.0753	0.1241				0.0473	7.7541
Lago-1	0.1353	0.1264	0.0089	N/A	0.0807	N/A				N/A	N/A
IP-Vdt-0	0.1121	0.1716	0.0595	0.2219	0.07997	0.14193				0.06513	1.094622
Baa-1	0.1331	0.1948	0.0617	0.233	0.05968	0.17332				0.09652	1.564344
IP-Smt-1	0.1177	0.1875	0.0698	0.2017	0.06298	0.13872				0.06192	0.887106

*F1 seed size data (mm^2^) is shown for F1 hybrids as well as the value for parent-of-origin effects. 2xA X 4xL (paternal-excess F1 hybrid triploids, ALL), 4xL X 2xA (maternal-excess F1 hybrid triploids, LLA). 2xA (diploid accession, AA), 2xL (diploid Ler-0, LL), 4xL (tetraploid Ler-0, LLLL), POE (Parent-of-origin effects, Δ), N/A, not available due to triploid block.*

Conversely, the F1 hybrids generated from the 2x L*er*-0 X 2x Accession cross direction showed a tendency toward WPH for seed size (Figure 3B and Supplementary Table 2). The highest levels of WPH were found in the L*er*-0/IP-Cad-0 and L*er*-0/IP-Vdt-0 F1 hybrids, with F1 seeds 16% smaller than the WP value. However, this pattern was more likely to be disrupted by the genome of the pollen donors, with numerous accessions such as Wank-2, Lu3-30, Tu-NK-12, Kus2-2, Copac-1, Ru4-16, DraIV 6–13, Kelsterbach-4, Bå1-2, Schl-7, Wt-5, Doubravnik7, Slavi-1, Gr-5, and Ta-0 producing Best-Parent and/or Mid-Parent Heterosis instead. For example, L*er*-0/Copac-1 and L*er*-0/Slavi-1 crosses produced F1 seeds that were more than 10% larger than their respective WP value. L*er*-0/Copac-1 F1 hybrids displayed BPH levels (BPH = 4.8%), while L*er*-0/Slavi-1 showed MPH levels (MPH = 2.4%). These parental effect results indicate that in these genotype combinations, the pollen donor genotype alters the heterotic response and suppresses the usual small-seeded “L*er*-0 mother effect.”

We also investigated whether there was any correlation between the genetic distance (or geolocation) between the tester L*er*-0 genotype and the accession genotypes used as either maternal or paternal parents in crosses with Ler-0. No correlation was observed between genetic distance (or geolocation) and heterosis effects on F1 seed size (Supplementary Figure 2 and Supplementary Table 6). We further investigated the broad-sense heritability of hybrid F1 seed size in relation to maternal and paternal parent of origin, where the maternal genotype explains 41.87% of the phenotypic variance in F1 hybrid seed size, whereas the paternal genotype explains 24.8% (Supplementary Table 7).

Overall, our results indicate that while F1 seed size heterosis is subject to natural variation within species, the extent of the heterosis effects are strongly influenced by parent-of-origin effects. While positive F1 seed size heterosis was typically associated with a L*er*-0 father effect, negative heterosis was generally induced by L*er*-0 maternal inheritance. However, there are inter-accession crosses which generate F1 seeds that do not conform with the general trends.

### Hybridity Enhances Parent-of-Origin Effects on F1 Seed Size in Inter-Ploidy Crosses Beyond Those Observed in Diploid Crosses

**FIGURE 4:**
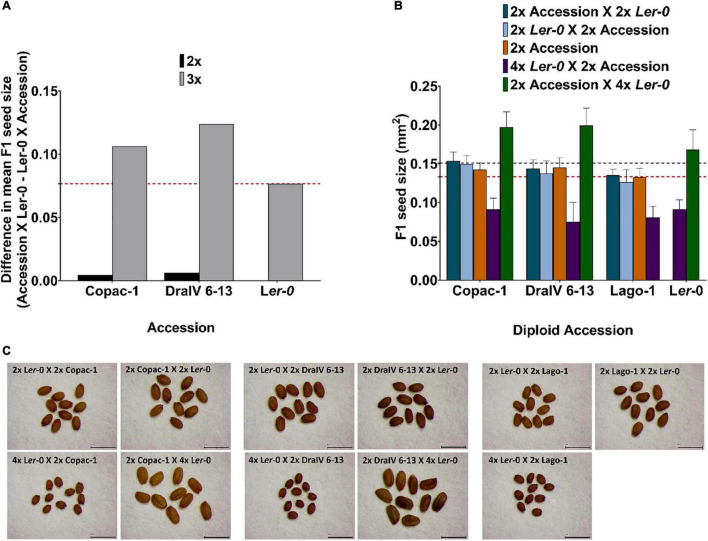
Hybridity enhances parent-of-origin effects in F1 triploids beyond that achieved at the diploid level. **(A)** Difference in F1 mean seed size for reciprocal isogenic (L*er*-0) and hybrid crosses at the diploid and triploid level. The red dashed line represents the parent-of-origin effects for L*er*-0 F1 isogenic triploids. **(B)** F1 hybrid seed size of balanced-ploidy crosses (2x Accession X 2x L*er*-0 and 2x L*er*-0 X 2x Accession) and inter-ploidy crosses (2x Accession X 4x L*er*-0 and 4x L*er*-0 X 2x Accession). Seed size of parental lines is also represented (orange for the natural accessions, a horizontal red dashed line for 2x L*er*-0 and black dashed line for 4x L*er*-0) for comparison with ploidy levels and their reciprocal F1 offspring. Error bars represent SD. **(C)** Seed size phenotypes of reciprocal F1 seeds in balanced and inter-ploidy crosses. Scale bar = 1mm.

To investigate whether parental genome dosage modifies the “L*er*-0 effect” observed across our reciprocal F1 hybrid diploid population, we repeated our crosses using a subset of the 71 accessions, but this time crossed to a tetraploid (4x) tester line, again in the L*er*-0 background. These genotypes were denoted “AA” for the diploid accession, “LLLL” for the tetraploid L*er*-0 tester, and “ALL” or “LLA” for the two sets of reciprocal F1 hybrid triploid seeds (using the genetically identical genotype of the embryo to indicate the two sets of offspring) (Supplementary Figure 1). We performed these crosses using the three accessions that displayed the weakest parent-of-origin effects on F1 seed size in diploid-diploid crosses (Copac-1, DraIV 6-13 and Lago-1) and those which displayed the strongest parental effects (IP-Vdt-0, Baa-1 and IP-Smt-1). Furthermore, reciprocal isogenic (genetically identical) inter-ploidy crosses between 2x and 4x L*er*-0 plants were also conducted to disaggregate heterosis effects due to genetic hybridity vs genome dosage (Figure 4 and Table 2). We have previously shown that these effects can interact in complex ways in control of plant biomass (Fort et al., 2016), and had hypothesised that similar interactions in control of seed size could help to explain later biomass accumulation.

Notably, we found that the “L*er*-0 effect” can be enhanced in pairs of reciprocal F1 hybrid triploid crosses of the accessions from the weakest parent-of-origin effects group. At the triploid level (3x), parent-of-origin effects in Copac-1/L*er*-0 hybrids increased up to Δ = 0.1062 mm^2^, in contrast to those of 2x level (Δ = 0.0045 mm^2^) (Table 2 and Figure 4A). To calculate parent-of-origin effects strictly associated with genetic hybridity, we subtracted the parent-of-origin effects caused by genome dosage observed in isogenic inter-ploidy reciprocal crosses of L*er*-0 (Δ = 0.0768 mm^2^) from that of Copac-1/L*er*-0 hybrid triploids (Δ = 0.1062 mm^2^). Strikingly, we found that genetic hybridity alone induced parent-of-origin effects that were 6.5 times higher in Copac-1/L*er*-0 hybrid triploids than in diploids (Table 2 and Figure 4A). Similarly, we detected parent-of-origin effects in DraIV 6-13/L*er*-0 hybrids triploids that were 7.75 times higher than those at the 2x level, enhancing the usual “L*er*-0 effect.” It was not possible to determine parent-of-origin effects at the 3x level in Lago-1/L*er*-0 reciprocal crosses, as paternal genome excess in this genotype cross combination leads to the postzygotic reproductive barrier known as the triploid block (Köhler et al., 2010, 2021). In contrast to these results, we observed that such “L*er*-0 effects” remained similar at both the 2x and 3x level in F1 hybrids of the accessions from the strongest parent-of-origin group (Table 2).

Overall, our results indicate that crosses between plants of unequal ploidy can drive additional hybridity-associated parent-of-origin effects in certain genotype combinations, even where no such parent-of-origin effects are seen in equivalent inter-accession crosses between diploids.

### Hybridity Has a Greater Effect on F1 Seed Size Heterosis at Triploid Level Compared to Diploid Level

To investigate whether the additional hybridity-associated parent-of-origin effects found in F1 hybrid triploids of L*er*-0 and the accessions Copac-1, DraIV 6-13 and Lago-1 modify the extent of heterosis, we calculated MPH levels at the 2x and 3x level. MPH ensures that both parental lines are considered in determining the level of heterosis in the F1 hybrid. For instance, a positive value for MPH may indicate that the F1 hybrid outperforms the average value of the parental lines, while a negative value indicates the hybrid falls below it. We therefore quantified the hybridity-specific contribution to deviations from the Mid-Parent value (MPV), beyond that achieved by genome dosage effects alone on 3x seed size.

**FIGURE 5:**
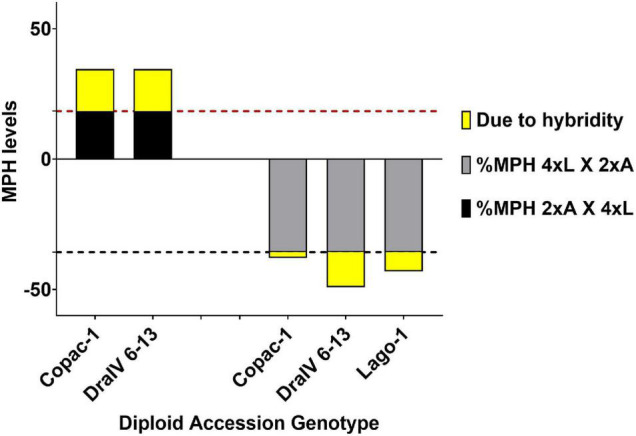
Hybridity has a greater effect on F1 seed size heterosis at the triploid level (3x). Mid-Parent Heterosis (MPH) levels in F1 3x seeds from reciprocal inter-ploidy crosses are shown. 2xA X 4xL (2x Accession X 4x L*er*-0), 4xL X 2xA (4x L*er*-0 X 2x Accession). The proportion due to hybridity is highlighted in yellow and it was calculated by subtracting the %MPH of reciprocal L*er*-0 F1 isogenic inter-ploidy crosses (18.4% for 2x4 crosses, represented by a horizontal red dashed line, and –35.7% for 4x2 crosses, represented by a horizontal black dashed line) from the MPH levels of F1 hybrid triploids.

On average, we found that hybridity alone increased F1 seed size by extra 17.9% in paternal-excess F1 triploids (3xp) and decreased it by 9.9% in maternal-excess F1 triploids (3xm) when compared to the L*er*-0 F1 isogenic 3x control (Supplementary Table 3). Despite the occurrence of MPH at the 2x level for certain cross combinations (Supplementary Table 4), we detected a greater deviation from the MPV in reciprocal inter-ploidy crosses (Supplementary Tables 3, 4), which exceeded both the relative levels of MPH achieved by polyploidy alone in L*er*-0 F1 isogenic triploids and those in F1 hybrid diploids (Supplementary Table 3). On one hand, F1 hybrid 3xp seeds were on average 16.1% larger than the MPV due to hybridity alone (after subtracting the MPH levels observed in L*er*-0 F1 isogenic 3x control crosses due to genome dosage), unlike their 2x counterparts which were only 5.5% larger. On the other hand, F1 hybrid 3xm seeds were 7.7% smaller than the MPV without considering genome dosage effects, in contrast to their hybrid diploid counterparts which were 0.8% larger (Figure 5 and Supplementary Table 3).

We also found that Copac-1 suppressed the “L*er*-0 mother effect,” as 2x L*er*-0 X 2x Copac-1 F1 seeds exceeded the MPV by 8.3% (Supplementary Table 3), while L*er*-0 mothers tended to generate smaller seeds (Figure 3). Paternal inheritance of Copac-1 increased F1 seed size independently of the ploidy level: i.e., F1 3xm derived from 4x L*er-0* X 2x Copac-1 crosses did not decrease F1 seed size beyond the genome dosage effect, unlike the other genotype combinations. Similarly, maternal contribution of Copac-1 also led to a F1 seed size increase in both 2x and 3x levels, although triploidy enhanced this positive effect on F1 seed size (Figure 5 and Supplementary Table 3). Finally, we sought to quantify the positive impact of paternal genome excess on F1 triploid seed size across 48 genetically distinct hybrids (Supplementary Figure 3 and Supplementary Table 5). On average, the introduction of hybridity at the 2x level increased seed size by 12.6% over the MPV. However, altering the paternal genome dosage of these F1 hybrids led to a further F1 seed size increase of 38.8%, where 20.4% of such enhancement was due to hybridity alone.

Overall, these findings indicate that hybridity has a greater effect than parental genome dosage effects on F1 seed size in a triploid context, and that genetic hybridity acts as a ‘heterosis enhancer’ under parental genome imbalance.

## Discussion

Heterosis effects on F1 seed size can occur in inter-accession crosses of *Arabidopsis*. Indeed, we and others have demonstrated pervasive parent-of-origin effects during plant reproduction (House et al., 2010). In Fort et al. (2016) we proposed such parental effects as causal to increased rosette biomass accumulation in polyploid F1 hybrids. In this study, we use a diversity panel of *Arabidopsis* accessions to (1) investigate natural variation in parent-of-origin effects on F1 seed size; (2) how parent-of-origin effects modify the heterosis response for F1 seed size; and (3) to identify “modifier” accessions which ablate or enhance parent-of-origin effects on F1 seed size. Here, we discuss our findings in relation to seed size control and heterosis effects in plant reproduction, breeding and biotechnology.

### Parent-of-Origin Effects Modify Paternal and Maternal Control of F1 Seed Size Heterosis in Hybrid Diploids

The *Arabidopsis* accession L*er*-0 generates small F1 seeds when used as a maternal (ovule) parent in inter-accession crosses, and larger ones when used as the paternal (pollen) parent. In this study we used L*er*-0 as a tester line on the basis of this property, as we have done previously (Duszynska et al., 2013, 2019), reasoning that, if this effect is robust across hybrid genotypes, it provides a basis for identifying those “modifier” accessions which enhance or suppress this heterosis effect, as well as for estimating the range of variation which this trait may display. We did indeed confirm that the expected pattern was generally robust in reciprocal crosses of 71 accessions, with F1 seeds produced using L*er*-0 as ovule parent being on average 0.82 times smaller their reciprocals (Figure 1A). This indicates that the trends reported from reciprocal crosses between L*er*-0/Accession such as Cvi by Alonso-Blanco et al. (1999), C24 by Zhu et al. (2016), and with groups of three or four accessions by House et al. (2010) and Groszmann et al. (2014) can be generalised across European and North American *Arabidopsis* accessions. Whether this generalised pattern holds true for crosses with more recently identified glacial relictual populations (Lee et al., 2017; Fulgione and Hancock, 2018; Toledo et al., 2020) or the poorly-understood African diversity of the species (Durvasula et al., 2017) remains to be determined.

Most studies on parental control of F1 seed size report pivotal roles for the maternal parent, especially in crops, where paternal effects are rarely reported (Singh et al., 2017; Baskin and Baskin, 2019). However, our data indicates significant paternal effects on F1 seed size heterosis in balanced-ploidy crosses. Indeed, we find that the maternal genotype explains 28% of the variation in F1 hybrid diploid seed size, similar to that of House et al. (2010), who found that maternal genotype explained 29.3% of the variation in F1 seed size in reciprocal hybrid diploid crosses. In contrast, we found that a further 22% of variation is contributed by the paternal genotype (*r* = 0.4658, *p* < 0.0001), considerably higher than the 10.4% estimated by House et al. and supporting our earlier supposition that paternal effects on seed size in *Arabidopsis thaliana* could lead to significant changes in biomass in mature plants (Fort et al., 2016). Furthermore, parent-of-origin effects due to genetic hybridity alone were on average 7.14 times higher in F1 hybrid triploids than those found in the diploid counterparts for the accessions from the weakest parent-of-origin effects group (Figure 4 and Table 2), while it remained similar at both the 2x and 3x level in F1 hybrids of the accessions from the strongest parent-of-origin group. These results suggest a hidden phenotypic plasticity in the “L*er*-0 effect” that can be modified by genome dosage.

### Differences in Mechanisms of Maternal and Paternal Control of F1 Seed Size

Notably, we could not find any significant correlation between the F1 seed size of pairs of reciprocal F1 hybrid diploids (Table 1), suggesting that the mechanisms for increases in F1 offspring seed size differ depending on whether the genome is inherited maternally or paternally. It is well-established that natural hybrid populations in both plants and animals tend to establish with one species consistently being the maternal parent, i.e., monodirectionally (Trier et al., 2014; Nieto Feliner et al., 2017). This may be due to ploidy differences that are only tolerated in the endosperm when in maternal excess (Donoghue et al., 2014; Köhler et al., 2021; Städler et al., 2021) or more rarely in paternal excess (Vallejo-Marín et al., 2016). However, in homoploid F1 hybrids, the basis of this bias remains unknown (except where maternal size is critical for gestation, as in mammals (Abbott et al., 2013). As F1 seed size is strongly associated with successful offspring establishment, differences in the genetic basis of seed size by parent-of-origin could provide a further explanation for such phenomena in plants.

Despite the critical importance of maternal control of seed size, we could not attribute the observed increase in size of F1 hybrid diploid seeds from 2x accession X 2x L*er*-0 crosses entirely to maternal effects. On average, the parental accessions used for reciprocal crosses had larger seed sizes than the tester line 2x L*er*-0, except for IP-Pro-0, Giffo-1, Np-0, Kyoto, Erg2-6, Gr-1, UII2-3, Toc-1, Fr-2, Tos-95-393, KNO1.37 and Lago-1. Despite the relatively smaller seed size of these exceptions, they produced larger F1 hybrid diploid seeds when used as maternal parent (Figure 1A). These results reveal a significant paternal effect of L*er*-0 that is independent of any effects derived as a female parent, as L*er*-0 tends to generate the biggest F1 seeds when used as paternal parent, irrespective of the maternal genotype, with only some exceptions (Supplementary Table 2 and Figure 3). The mechanisms for this “L*er*-effect” remain unknown but likely involve increased seed sink strength: this has been argued as a likely role for imprinted Paternally Expressed Genes (iPEGs) (Baroux et al., 2002; McKeown et al., 2013b), which we have shown to be preferentially subject to positive selection in *Arabidopsis* (Tuteja et al., 2019), although direct evidence for iPEGs controlling sink strength in this species is lacking.

We also found that parent-of-origin effects on F1 hybrid seed size are widely influenced by natural variation (Figure 1B). We detect genotype combinations that lead to both strong and weak parent-of-origin effects on F1 seeds in homoploid crosses. Such reciprocal F1 hybrid diploid seeds are composed of embryos who are genetically identical (but epigenetically different), but which also differ in their seed coat composition which is of maternal origin. Similarly, the endosperm genome in F1 seeds may act as a source of variation, as it differs in the genome dosage composition (2m:1p maternal:paternal genome ratio), potentially resulting in unequal parental genome contribution effects that may explain the phenotypic F1 seed size differences we observe (Lafon-Placette and Köhler, 2014; Pignatta et al., 2014; Lafon Placette, 2020; Picard et al., 2021). Another possibility is that parent-of-origin effects in F1 hybrid seeds can alter cyto-nuclear interactions that contribute to F1 seed size heterosis (Flood et al., 2020), ultimately altering the F1 hybrid’s transcriptional program (Botet and Keurentjes, 2020). Flood et al. (2020) have revealed how organellar genomes affect seed size through new plasmotype–nucleotype combinations (cybrids) across five genetically different reciprocal cybrids of *Arabidopsis*. In particular, a seed size increment (1.6× larger), in comparison with its nuclear parent, was found in L*er*-0/Sha cybrids (L*er*-0 nucleotype, Sha plasmotype), suggesting that cyto-nuclear interactions are important for seed size heterosis and may be altered in F1 hybrids (Flood et al., 2020).

We did not detect any significant correlation between differences in mean F1 seed size of parental lines and that of reciprocal F1 hybrid diploids. Hence, similar seed size in the parental lines does not necessarily lead to similar F1 size in reciprocal F1 hybrid diploids. This was particularly apparent in reciprocal crosses of 2x L*er*-0 with some accessions (e.g., Toc-1, Fr-2, UII2-3, Tu-NK-12, Tos-95-393, IP-Vdt-0, and Baa-1), where the parental seed size was very similar to diploid L*er*-0, but the F1 hybrid seeds differed considerably (Figures 1, 2).

### Seed Size Heterosis Depends on Parent-of-Origin Effects

We detected significant natural variation in the extent of the heterosis effects on F1 seed size when diploid accessions were reciprocally crossed, which depended on the cross direction (Figure 3 and Table 1). BPH compares the F1 hybrid trait values only to the best performing parent, in contrast to MPH, which is a relative measure that ensures that both parental lines are considered in determining the level of heterosis in the F1 hybrid. As the F1 hybrid populations share a common parent (2x L*er*-0), we could attribute the differences in MPH levels to the differences across the different natural accessions used as either maternal or paternal parent. Consistent with our previous observations on L*er*-0 fathers (pollen) generating bigger F1 hybrid seeds, high levels of BPH were found across 2x Accession X 2x L*er*-0 F1 hybrid population except for the F1 hybrids derived from the accessions DraIV-6-13, Duk, Zdrl 2-21, Ei-2 and IP-Pro-0, whose F1 seeds instead displayed MPH.

BPH and WPH for F1 seed size are indicative of extreme or transgressive phenotypes (Rieseberg et al., 1999; Mackay et al., 2021) and can be of agricultural importance when genotype combinations lead to larger (BPH) or smaller seeds (WPH), depending on the desired seed size for certain species. WPH can be considered an inverse of conventional heterosis and is typically hypothesised to be due to outbreeding depression, potentially derived from negative epistatic interactions (Bomblies et al., 2007; Oakley et al., 2015). In this case, hybridisation would disrupt favourable allele interactions (Burke and Arnold, 2001; Bomblies et al., 2007; Alcázar et al., 2010; Chae et al., 2014; Chen et al., 2016; Boeven et al., 2020). We found genotype combinations that led to transgressive phenotypes in both directions, which confirm the non-additive mode of inheritance typical of heterosis.

The *Arabidopsis* accessions used in this study covered a wide geographic distribution (Supplementary Table 1) and showed wide natural variation for heterosis effects on F1 seed size. It is unclear whether the extent of heterosis can increase with genetic distance between parents, with most studies failing to support such correlations (Schnable and Springer, 2013; Seymour et al., 2016; Labroo et al., 2021), consistent with our results in this study (Supplementary Figure 2). Furthermore, we find no significant association between geolocation (latitude and longitude) and phenotypic variance for parent-of-origin effects, reciprocal F1 hybrid diploid seed size or seed size of selfed isogenic parental lines (Supplementary Table 6). Our results are in agreement with Ren et al. (2019), where no significant association between geographical distribution of 191 accessions and seed size from selfed plants was found. Our heritability analysis indicates that the maternal genotype explains 41.87% of the phenotypic variance in F1 hybrid seed size, whereas the paternal genotype explains 24.8% (Supplementary Table 7). These results are consistent with our findings in Table 1 that show a higher correlation between maternal seed size and its F1 hybrid seed offspring in a 2x Accession X 2x Ler-0 cross than the opposite 2x Ler-0 X 2x Accession cross.

### Hybridity Enhances the “L*er*-0 Effect” on F1 Hybrid Triploids

Inter-ploidy crosses alter the genome dosage balance ratio in the endosperm (and the timing of endosperm cellularisation), leading to developmental effects on F1 seed size. Many genes can be dosage-sensitive, due to genomic imprinting (Batista and Köhler, 2020), stoichiometric imbalances in macromolecular complexes (Veitia and Birchler, 2015), or other dosage sensitivities (Birchler et al., 2005), as well as possible maternal-zygotic crosstalk mechanisms (Doughty et al., 2014; Lafon-Placette and Köhler, 2016; Robert et al., 2018). Several studies suggest dosage-dependent phenotypes can also contribute to heterosis (Birchler and Veitia, 2012; Birchler et al., 2016). For instance, the major Quantitative Trait Loci (QTL) in tomato *fruit weight 2.2* (*fw2.2*) displays a dosage-dependent heterotic response, as higher levels of gene expression correlated negatively with smaller fruit size (Cong et al., 2002; Liu et al., 2003). Furthermore, *SINGLE FLOWER TRUSS* (*SFT*) gene in tomato generates yield heterosis only when the loss-of-function allele is heterozygous, consistent with dosage-sensitivity (Krieger et al., 2010; McKeown et al., 2013a). Our results indicate that effects such as these may be revealed by parental genome dosage imbalance, and that these effects can enhance hybridity-associated parent-of-origin effects. Triploidy and hybridity can therefore generate unique phenotypes and potentially reveal cryptic genetic variation and/or interactions that are hidden in euploids and/or isogenics (Pires et al., 2016; Tabib et al., 2016).

## Conclusion

In this study we have quantified natural variation in parent-of-origin effects on F1 seed size in the model plant, *Arabidopsis*, in both homoploid diploid and inter-ploidy (tetraploid X diploid) crosses. Our results indicates that parental effects can strongly influence F1 seed size, with a much larger influence of the paternal genome on F1 seed size than previously appreciated. Parent-of-origin effects on seed phenotypes in angiosperms can be sporophytic or gametophytic (Spillane et al., 2002). While maternal effects on seed size can have a sporophytic (e.g., seed coat tissues) and/or gametophytic (e.g., egg cell, central cell) basis, paternal effects are most likely to be gametophytic effects. The underlying molecular mechanisms responsible for the significant maternal and paternal effects on F1 seed size we identify in this study remain to be identified. Our findings have implications for hybrid seed biology and may open new avenues for the study of paternal contribution to F1 seed size, potentially generating new applications in plant breeding programs. The lack of correlation between maternal and paternal effects indicates that both the genetic pathways mediated, and the evolutionary pressures likely to act on them, differ between pathways for maternal and paternal control of F1 seed size. We also demonstrate that genetic hybridity alone can enhance parent-of-origin effects over seven-fold in F1 hybrids generated from inter-ploidy crosses, indicating that genetic hybridity effects can be genetically enhanced by parental genome dosage effects. We conclude that the interaction between the effects of hybridity and parental genome dosage need to be considered for more complete understanding of the mechanisms underpinning heterosis effects.

## Data Availability Statement

The original contributions presented in the study are included in the article/Supplementary Material, further inquiries can be directed to the corresponding author.

## Author Contributions

CS and RC-B planned and designed the research. RC-B and AF performed the experiments. RC-B and RC analysed the data. CS, RC-B, PM, and GB drafted the manuscript, which was reviewed and revised by all authors.

## Conflict of Interest

The authors declare that the research was conducted in the absence of any commercial or financial relationships that could be construed as a potential conflict of interest.

## Publisher’s Note

All claims expressed in this article are solely those of the authors and do not necessarily represent those of their affiliated organizations, or those of the publisher, the editors and the reviewers. Any product that may be evaluated in this article, or claim that may be made by its manufacturer, is not guaranteed or endorsed by the publisher.

## Supplementary Material

Supplementary Figure 1 Illustration of the crossing scheme used and schematic diagram of the ploidy level of the different F1 seed compartments from inter-ploidy (unbalanced) crosses. Chromosomes of the tetraploid L*er*-0 tester line (genotype LLLL) are represented in black, while those of Copac-1, DraIV 6-13, Lago-1, IP-Vdt-0, Baa-1 and IP-Smt-1 (genotype AA) are shown in gray.

Supplementary Figure 2 Correlation between the parent-of-origin effects of the 71 accessions on F1 hybrid seed size when crossed with the tester Line Ler-0, and the genetic distance between these accessions and the reference accession L*er*-0. The red line shows the regression of parent-of-origin effects onto the genetic distance, to which the R^2^ value and p-value correspond.

Supplementary Figure 3 Seed size (mm^2^) of paternal-excess F1 hybrid triploids using 48 genetically different maternal genotypes that are part of the accession panel used in this study. The horizontal red dashed line represents the mean seed size value for the L*er*-0 F1 isogenic triploid control (0.1682 mm^2^). Error bars represent SD, N > 20 seeds/genotype.

Supplementary Table 1 List of *Arabidopsis thaliana* natural diploid accessions used in this study and their geolocation.

Supplementary Table 2 Calculations of Best-Parent (%BPH), Mid-Parent (%MPH) and Worst-Parent Heterosis (%WPH) for F1 hybrid diploid seed size. Values that do not follow the observed trend are highlighted in red.

Supplementary Table 3 MPH levels of reciprocal balanced (2x X 2x) and inter-ploidy hybrid crosses (2x X 4x, 4x X 2x). 2xA (diploid accession), 2xL (2x L*er*-0), 4xL (4x L*er*-0). N/A, not available due to triploid block.

Supplementary Table 4 Deviations from the mid-parent value (MPV) in F1 hybrid diploids and triploids. Data represents the mean F1 seed size. Asterisks represent significant differences between the F1 hybrids and the MPV (‘^****^’ and ‘*’ indicate statistical significance at *p*-value < 0.0001 and *p*-value < 0.05, respectively, determined by Student’s *t*-test. *Ns*, not significant). N/A, not available due to triploid block.

Supplementary Table 5 MPH levels across 48 genetically different F1 hybrids at the diploid and paternal-excess triploid level. 2xA (diploid accession), 2xL (2x L*er*-0), 4xL (4x L*er*-0).

Supplementary Table 6 Influence of geolocation on phenotypic variation. Pearson correlation coefficient (r) was used to determine the latitudinal and longitudinal association of the 71 genetically different accessions of this study with reciprocal F1 hybrid diploid seed size, seed size of selfed isogenic diploid parental lines and parent-of-origin effects. Correlation is significant at the 0.05 level.

Supplementary Table 7 Broad-sense heritability of hybrid F1 seed size in terms of the parent of origin.

## Abbreviations

*Arabidopsis*: *Arabidopsis thaliana*
POE: parent-of-origin effects
2x: diploid
4x: tetraploid
3xm: maternal-excess F1 triploids
3xp: paternal-excess F1 triploids
BPH: Best-Parent Heterosis levels
MPH: Mid-Parent Heterosis levels
WPH: Worst-Parent Heterosis levels
MPV: mid-parent value.
