# Supplementary figures and images for: Parent-of-Origin Effects on Seed Size Modify Heterosis Responses in Arabidopsis thaliana
Source: Front Plant Sci. 2022 Mar 7;13:835219. doi: 10.3389/fpls.2022.835219 (PMC8940307; doi:10.3389/fpls.2022.835219)

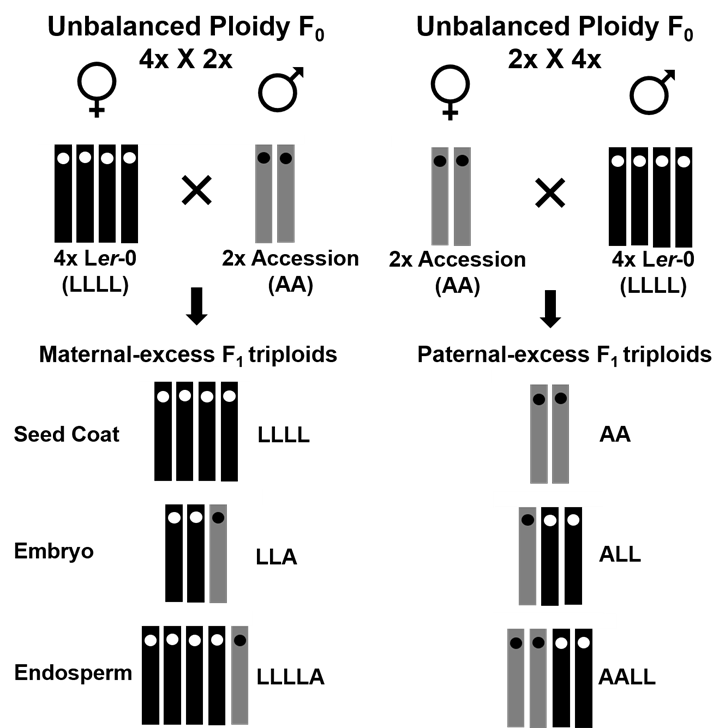

Supplement: Supplementary Figure 1 — Illustration of the crossing scheme used and schematic diagram of the ploidy level of the different F1 seed compartments from inter-ploidy (unbalanced) crosses. Chromosomes of the tetraploid Ler-0 tester line (genotype LLLL) are represented in black, while those of Copac-1, DraIV 6-13, Lago-1, IP-Vdt-0, Baa-1 and IP-Smt-1 (genotype AA) are shown in gray. [file Image_1.TIF]

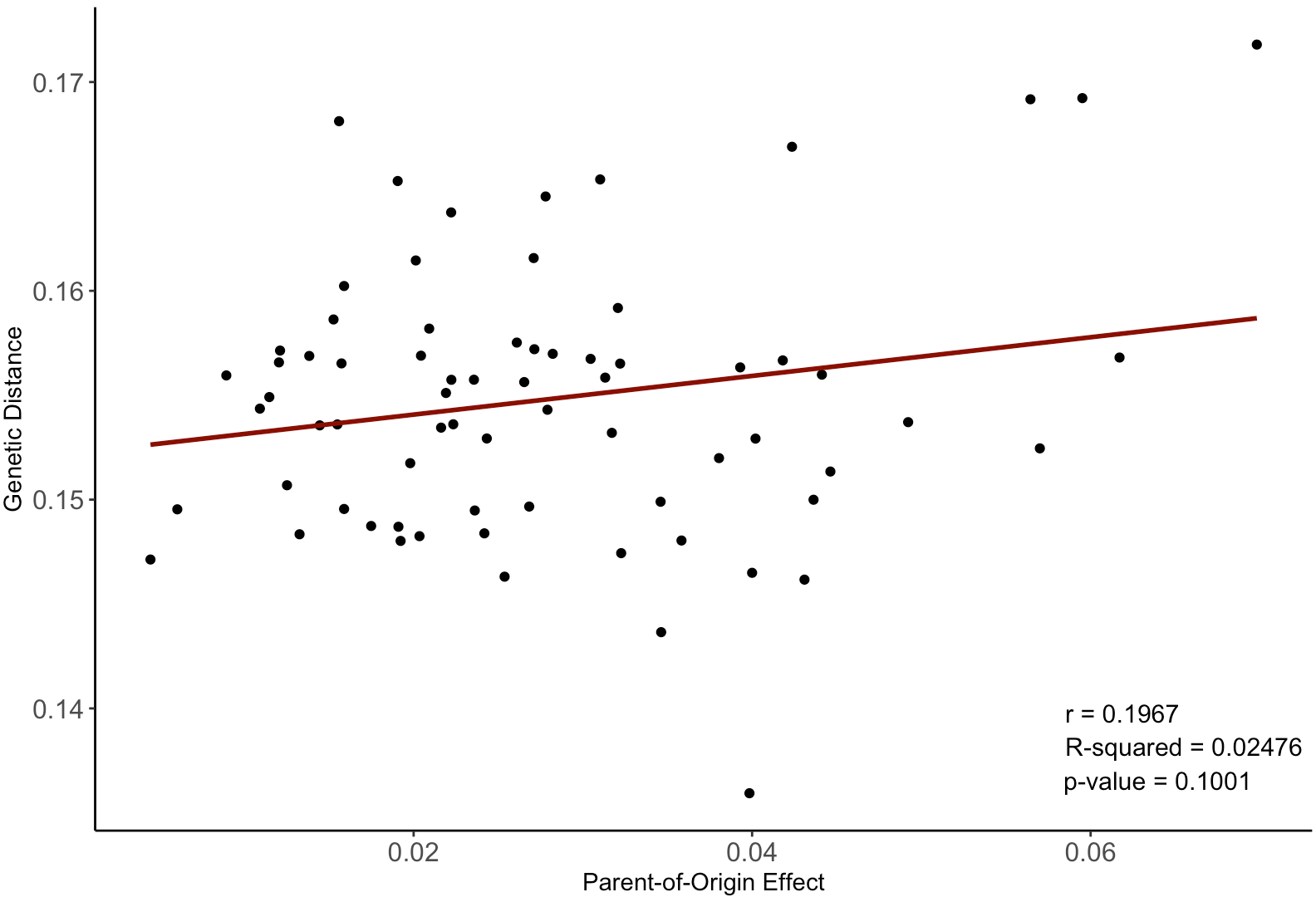

Supplement: Supplementary Figure 2 — Correlation between the parent-of-origin effects of the 71 accessions on F1 hybrid seed size when crossed with the tester Line Ler-0, and the genetic distance between these accessions and the reference accession Ler-0. The red line shows the regression of parent-of-origin effects onto the genetic distance, to which the R2 value and p-value correspond. [file Image_2.TIFF]

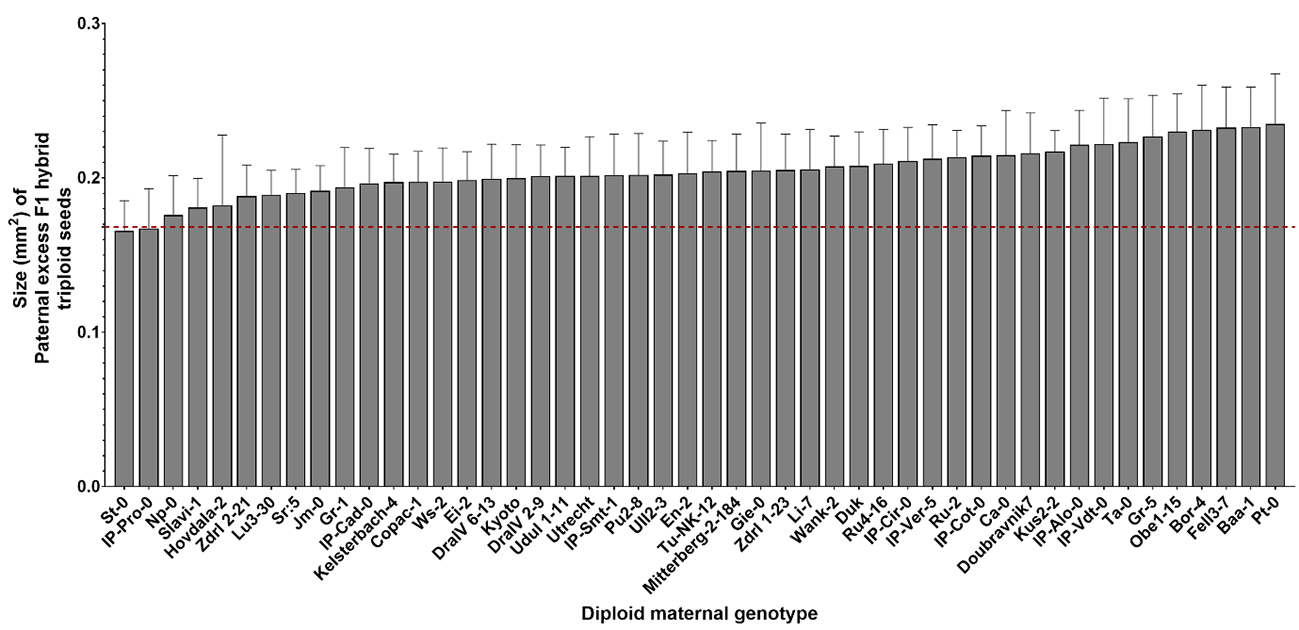

Supplement: Supplementary Figure 3 — Seed size (mm2) of paternal-excess F1 hybrid triploids using 48 genetically different maternal genotypes that are part of the accession panel used in this study. The horizontal red dashed line represents the mean seed size value for the Ler-0 F1 isogenic triploid control (0.1682 mm2). Error bars represent SD, N > 20 seeds/genotype. [file Image_3.TIF]
