# Supplementary material for: Parent-of-Origin Effects on Seed Size Modify Heterosis Responses in Arabidopsis thaliana
Source: Front Plant Sci. 2022 Mar 7;13:835219. doi: 10.3389/fpls.2022.835219 (PMC8940307; doi:10.3389/fpls.2022.835219)
Supplement: Supplementary Table 1 — List of Arabidopsis thaliana natural diploid accessions used in this study and their geolocation. [file Table_1.DOCX]

| Name | Country | Latitude | Longitude | 1001genomesID |
| --- | --- | --- | --- | --- |
| Doubravnik7 | CZE | 49.4211 | 16.3497 | 410 |
| Gr-1 | AUT | 47 | 15.5 | 430 |
| Tos-95-393 | SWE | 59.4333 | 17.0167 | 1257 |
| DraIV 2-9 | CZE | 49.4112 | 16.2815 | 5907 |
| DraIV 6-13 | CZE | 49.4112 | 16.2815 | 5984 |
| Duk | CZE | 49.1 | 16.2 | 6008 |
| Eds-1 | SWE | 62.9 | 18.4 | 6016 |
| Hovdala-2 | SWE | 56.1 | 13.74 | 6039 |
| UduI 1-11 | CZE | 49.2771 | 16.6314 | 6296 |
| ZdrI 1-23 | CZE | 49.3853 | 16.2544 | 6424 |
| ZdrI 2-21 | CZE | 49.3853 | 16.2544 | 6445 |
| Bor-4 | CZE | 49.4013 | 16.2326 | 6903 |
| Ei-2 | GER | 50.3 | 6.3 | 6915 |
| Pu2-8 | CZE | 49.42 | 16.36 | 6957 |
| Ull2-3 | SWE | 56.0648 | 13.9707 | 6973 |
| Ws-2 | RUS | 52.3 | 30 | 6981 |
| Wt-5 | GER | 52.3 | 9.3 | 6982 |
| Ak-1 | GER | 48.0683 | 7.62551 | 6987 |
| Appt-1 | NED | 51.8333 | 5.5833 | 6997 |
| Baa-1 | NED | 51.3333 | 6.1 | 7002 |
| Ca-0 | GER | 50.2981 | 8.26607 | 7062 |
| En-2 | GER | 50 | 8.5 | 7119 |
| Fr-2 | GER | 50.1102 | 8.6822 | 7133 |
| Gie-0 | GER | 50.584 | 8.67825 | 7147 |
| Gr-5 | AUT | 47 | 15.5 | 7158 |
| Jm-0 | CZE | 49 | 15 | 7177 |
| Kyoto | JPN | 35.0085 | 135.752 | 7207 |
| Li-7 | GER | 50.3833 | 8.0666 | 7231 |
| Mnz-0 | GER | 50.001 | 8.26664 | 7244 |
| Mh-0 | GER | 50.95 | 7.5 | 7255 |
| Np-0 | GER | 52.6969 | 10.981 | 7268 |
| Pt-0 | GER | 53.476 | 10.6065 | 7305 |
| Ta-0 | CZE | 49.5 | 14.5 | 7349 |
| Utrecht | NED | 52.0918 | 5.1145 | 7382 |
| Ws-0.2 | RUS | 52.3 | 30 | 7396 |
| KNO1.37 | USA | 41.273 | -86.625 | 7717 |
| Bå1-2 | SWE | 56.4 | 12.9 | 8256 |
| Rak-2 | CZE | 49 | 16 | 8365 |
| Sr:5 | SWE | 58.9 | 11.2 | 8386 |
| St-0 | SWE | 59 | 18 | 8387 |
| Kelsterbach-4 | GER | 50.0667 | 8.5333 | 8420 |
| IP-Alo-0 | POR | 40.11 | -7.47 | 9506 |
| IP-Cad-0 | ESP | 40.37 | -5.74 | 9527 |
| IP-Pro-0 | ESP | 43.28 | -6.01 | 9571 |
| IP-Vad-0 | ESP | 42.86 | -3.59 | 9591 |
| IP-Vdt-0 | ESP | 40.89 | -5.5 | 9595 |
| IP-Ver-5 | ESP | 41.95 | -7.45 | 9596 |
| Giffo-1 | ITA | 38.44 | 16.13 | 9653 |
| Marce-1 | ITA | 38.92 | 16.47 | 9655 |
| Nicas-1 | ITA | 38.97 | 16.34 | 9658 |
| Mitterberg-2-184 | ITA | 46.37 | 11.28 | 9668 |
| Toc-1 | ROU | 46.01 | 22.33 | 9739 |
| Ru4-16 | GER | 48.57 | 9.16 | 9768 |
| Hof-1 | GER | 48.41 | 8.85 | 9772 |
| Fell3-7 | GER | 48.43 | 8.79 | 9776 |
| Bach-7 | GER | 48.41 | 8.84 | 9778 |
| Kus2-2 | GER | 48.52 | 9.11 | 9781 |
| Lu3-30 | GER | 48.53 | 9.09 | 9782 |
| Erg2-6 | GER | 48.5 | 8.8 | 9784 |
| Wank-2 | GER | 48.5 | 9.11 | 9795 |
| Obe1-15 | GER | 48.45 | 8.87 | 9804 |
| Ru-2 | GER | 48.56 | 9.16 | 9806 |
| Schl-7 | GER | 48.6 | 9.22 | 9807 |
| Tu-NK-12 | GER | 48.52 | 9.05 | 9811 |
| IP-Cir-0 | ESP | 40.61 | -6.57 | 9835 |
| IP-Cot-0 | ESP | 41.83 | -5.38 | 9838 |
| IP-Smt-1 | ESP | 40.95 | -5.63 | 9897 |
| Lago-1 | ITA | 39.18 | 16.26 | 9963 |
| Altenb-2 | ITA | 46.37 | 11.24 | 9970 |
| Slavi-1 | BUL | 41.43 | 23.65 | 9985 |
| Copac-1 | ROU | 46.11 | 21.95 | 10005 |
| L*er*-0 | GER | 47.984 | 10.8719 | 7213 |
