# Supplementary material for: Parent-of-Origin Effects on Seed Size Modify Heterosis Responses in Arabidopsis thaliana
Source: Front Plant Sci. 2022 Mar 7;13:835219. doi: 10.3389/fpls.2022.835219 (PMC8940307; doi:10.3389/fpls.2022.835219)
Supplement: Supplementary Table 2 — Calculations of Best-Parent (%BPH), Mid-Parent (%MPH) and Worst-Parent Heterosis (%WPH) for F1 hybrid diploid seed size. Values that do not follow the observed trend are highlighted in red. [file Table_2.DOCX]

| Diploid  Accession  Name | %BPH Accession X  2x *Ler-0* | %MPH Accession X  2x Ler-0 | %WPH Accession X  2x Ler-0 | %BPH  2x *Ler-0*  X Accession | %MPH  2x Ler-0  X  Accession | %WPH 2x Ler-0  X Accession |
| --- | --- | --- | --- | --- | --- | --- |
| IP-Pro-0 | 0.0000 | 6.0932 | 12.9771 | -14.3393 | -9.1199 | -3.2231 |
| Giffo-1 | 3.8288 | 9.5880 | 16.0235 | -11.2613 | -6.3391 | -0.8389 |
| Np-0 | 8.1081 | 13.9691 | 20.5021 | -10.8859 | -6.0546 | -0.6695 |
| Kyoto | 7.5075 | 11.4397 | 15.6704 | -7.7327 | -4.3580 | -0.7270 |
| Erg2-6 | 13.8138 | 16.9302 | 20.2220 | -7.3574 | -4.8207 | -2.1412 |
| Gr-1 | 8.0330 | 10.7775 | 13.6651 | -10.1351 | -7.8522 | -5.4502 |
| Ull2-3 | 18.0180 | 20.8766 | 23.8771 | -15.0901 | -13.0334 | -10.8747 |
| Toc-1 | 17.7177 | 19.1037 | 20.5227 | -14.6396 | -13.6346 | -12.6057 |
| Fr-2 | 17.4174 | 18.1269 | 18.8450 | -5.4805 | -4.9094 | -4.3313 |
| Tos-95-393 | 34.8348 | 35.3429 | 35.8548 | -2.1021 | -1.7332 | -1.3616 |
| KNO1.37 | 1.9520 | 2.2976 | 2.6455 | -9.5345 | -9.2279 | -8.9191 |
| Lago-1 | 1.5766 | 1.8442 | 2.1132 | -5.1051 | -4.8551 | -4.6038 |
| Ws-0.2 | 1.7164 | 2.0210 | 2.3273 | -13.0597 | -12.7994 | -12.5375 |
| Ws-2 | 6.3291 | 6.7664 | 7.2072 | -9.8287 | -9.4579 | -9.0841 |
| IP-Ver-5 | 6.4112 | 7.4005 | 8.4084 | -5.3058 | -4.4254 | -3.5285 |
| Nicas-1 | 0.8843 | 1.8222 | 2.7778 | -10.6116 | -9.7806 | -8.9339 |
| Sr:5 | 9.3382 | 10.4755 | 11.6366 | -2.3529 | -1.3373 | -0.3003 |
| Mnz-0 | 1.4588 | 2.9227 | 4.4294 | -10.0656 | -8.7680 | -7.4324 |
| Wank-2 | 5.5797 | 7.4484 | 9.3844 | -3.4783 | -1.7699 | 0.0000 |
| Mh-0 | 7.0188 | 8.9904 | 11.0360 | -8.8278 | -7.1481 | -5.4054 |
| Lu3-30 | 14.1823 | 16.2859 | 18.4685 | -0.3618 | 1.4738 | 3.3784 |
| DraIV 2-9 | 7.5090 | 9.6062 | 11.7868 | -11.3357 | -9.6062 | -7.8078 |
| Ca-0 | 4.4043 | 6.4409 | 8.5586 | -5.1986 | -3.3493 | -1.4264 |
| Rak-2 | 5.8399 | 7.9809 | 10.2102 | -13.4823 | -11.7323 | -9.9099 |
| IP-Cir-0 | 3.4483 | 5.7269 | 8.1081 | -18.8218 | -17.0338 | -15.1652 |
| IP-Cad-0 | 9.4729 | 12.3538 | 15.3904 | -20.7265 | -18.6404 | -16.4414 |
| Pu2-8 | 12.0285 | 15.0164 | 18.1682 | -12.5979 | -10.2667 | -7.8078 |
| Baa-1 | 38.1560 | 42.0861 | 46.2462 | -5.6028 | -2.9176 | -0.0751 |
| Ak-1 | 1.1323 | 4.1166 | 7.2823 | -7.4310 | -4.6995 | -1.8018 |
| Tu-NK-12 | 26.4311 | 30.2512 | 34.3093 | -5.0883 | -2.2206 | 0.8258 |
| Obe1-15 | 11.0876 | 14.4833 | 18.0931 | -7.6271 | -4.8035 | -1.8018 |
| Fell3-7 | 19.4072 | 23.0993 | 27.0270 | -10.0917 | -7.3117 | -4.3544 |
| St-0 | 0.6338 | 3.8517 | 7.2823 | -7.0423 | -4.0698 | -0.9009 |
| Utrecht | 0.3519 | 3.5961 | 7.0571 | -14.3561 | -11.5874 | -8.6336 |
| Kus2-2 | 13.2022 | 16.9811 | 21.0210 | -3.8624 | -0.6531 | 2.7778 |
| Copac-1 | 8.0056 | 11.6110 | 15.4655 | 4.8455 | 8.3454 | 12.0871 |
| Appt-1 | 7.3684 | 10.9902 | 14.8649 | -8.2807 | -5.1868 | -1.8769 |
| Jm-0 | 0.6272 | 4.3730 | 8.4084 | -12.7526 | -9.5049 | -6.0060 |
| Li-7 | 8.8440 | 12.9335 | 17.3423 | -10.0975 | -6.7197 | -3.0781 |
| Ru4-16 | 1.8776 | 5.7762 | 9.9850 | -6.5369 | -2.9603 | 0.9009 |
| UduI 1-11 | 3.2526 | 7.4541 | 12.0120 | -19.1003 | -15.8084 | -12.2372 |
|  |  |  |  |  |  |  |
|  |  |  |  |  |  |  |
| ZdrI 1-23 | 7.9475 | 12.4145 | 17.2673 | -15.9641 | -12.4865 | -8.7087 |
| Gie-0 | 21.2517 | 26.5614 | 32.3574 | -17.9505 | -14.3575 | -10.4354 |
| DraIV 6-13 | -1.1684 | 3.1934 | 7.9580 | -5.3608 | -1.1841 | 3.3784 |
| Hovdala-2 | 8.0082 | 12.9968 | 18.4685 | -19.2334 | -15.5030 | -11.4114 |
| IP-Alo-0 | 18.7286 | 24.2934 | 30.4054 | -19.8223 | -16.0644 | -11.9369 |
| Duk | -0.6807 | 4.1771 | 9.5345 | -16.7461 | -12.6740 | -8.1832 |
| IP-Vdt-0 | 16.5761 | 22.3966 | 28.8288 | -23.8451 | -20.0428 | -15.8408 |
| Marce-1 | 10.9301 | 16.5062 | 22.6727 | -15.7502 | -11.5152 | -6.8318 |
| Eds-1 | 3.2498 | 8.5796 | 14.4895 | -11.7806 | -7.2268 | -2.1772 |
| Kelsterbach-4 | 1.1494 | 6.4390 | 12.3123 | -8.6545 | -3.8776 | 1.4264 |
| Hof-1 | 7.8841 | 13.7074 | 20.1952 | -13.2749 | -8.5938 | -3.3784 |
| IP-Cot-0 | 1.0738 | 6.7328 | 13.0631 | -17.5839 | -12.9695 | -7.8078 |
| Ru-2 | 3.8102 | 9.8303 | 16.5916 | -11.9652 | -6.8600 | -1.1261 |
| Bå1-2 | 10.1064 | 16.7842 | 24.3243 | -11.3032 | -5.9238 | 0.1502 |
| Bach-7 | 1.5957 | 7.7574 | 14.7147 | -16.9548 | -11.9182 | -6.2312 |
| ZdrI 2-21 | -0.3981 | 5.7415 | 12.6877 | -12.0106 | -6.5868 | -0.4505 |
| IP-Vad-0 | 1.0610 | 7.3239 | 14.4144 | -16.9098 | -11.7606 | -5.9309 |
| Ei-2 | -4.5665 | 1.4421 | 8.2583 | -14.8246 | -9.4618 | -3.3784 |
| Schl-7 | 14.9934 | 22.3472 | 30.7057 | -6.1427 | -0.1405 | 6.6817 |
| Wt-5 | 1.0492 | 7.8754 | 15.6907 | -6.0984 | 0.2450 | 7.5075 |
| Bor-4 | 5.5701 | 12.7362 | 20.9459 | -20.6422 | -15.2554 | -9.0841 |
| En-2 | 4.7557 | 12.1730 | 20.7207 | -20.0651 | -14.4053 | -7.8829 |
| Mitterberg-2-184 | 4.7557 | 12.1730 | 20.7207 | -15.8958 | -9.9407 | -3.0781 |
| Doubravnik7 | 2.9183 | 10.4384 | 19.1441 | -9.4682 | -2.8532 | 4.8048 |
| IP-Smt-1 | 18.5958 | 28.7333 | 40.7658 | -25.5534 | -19.1898 | -11.6366 |
| Altenb-2 | 2.2542 | 11.5056 | 22.5976 | -20.1628 | -12.9396 | -4.2793 |
| Slavi-1 | 0.5573 | 10.2138 | 21.9219 | -6.5635 | 2.4092 | 13.2883 |
| Gr-5 | 1.3317 | 12.1984 | 25.6757 | -12.1671 | -2.7480 | 8.9339 |
| Pt-0 | 2.2129 | 13.7816 | 28.3033 | -21.8301 | -12.9827 | -1.8769 |
| Ta-0 | 2.3021 | 17.0575 | 36.7868 | -22.1786 | -10.9541 | 4.0541 |
