# Supplementary material for: Parent-of-Origin Effects on Seed Size Modify Heterosis Responses in Arabidopsis thaliana
Source: Front Plant Sci. 2022 Mar 7;13:835219. doi: 10.3389/fpls.2022.835219 (PMC8940307; doi:10.3389/fpls.2022.835219)
Supplement: Supplementary Table 5 — MPH levels across 48 genetically different F1 hybrids at the diploid and paternal-excess triploid level. 2xA (diploid accession), 2xL (2x Ler-0), 4xL (4x Ler-0). [file Table_5.DOCX]

| Accession | %MPH 2xA X 4xL | %MPH 2xA X 2xL |
| --- | --- | --- |
| Baa-1 | 59.6437 | 42.0861 |
| Bor-4 | 52.2900 | 12.7362 |
| Ca-0 | 48.3760 | 6.4409 |
| Copac-1 | 34.5380 | 11.6110 |
| Doubravnik7 | 41.5929 | 10.4384 |
| DraIV 2-9 | 38.9772 | 9.6062 |
| DraIV 6-13 | 34.5479 | 3.1934 |
| Duk | 39.4896 | 4.1771 |
| Ei-2 | 31.5232 | 1.4421 |
| En-2 | 33.3771 | 12.1730 |
| Fell3-7 | 58.9884 | 23.0993 |
| Gie-0 | 38.1033 | 26.5614 |
| Gr-1 | 39.7477 | 10.7775 |
| Gr-5 | 43.5622 | 12.1984 |
| Hovdala-2 | 22.6936 | 12.9968 |
| IP-Alo-0 | 49.0579 | 24.2934 |
| IP-Cad-0 | 34.9125 | 12.3538 |
| IP-Cir-0 | 45.4671 | 5.7269 |
| IP-Cot-0 | 43.0477 | 6.7328 |
| IP-Pro-0 | 24.4792 | 6.0932 |
| IP-Smt-1 | 30.5502 | 28.7333 |
| IP-Vdt-0 | 48.8762 | 22.3966 |
| IP-Ver-5 | 48.2205 | 7.4005 |
| Jm-0 | 30.1630 | 4.3730 |
| Kelsterbach-4 | 31.9946 | 6.4390 |
| Kus2-2 | 48.0395 | 16.9811 |
| Kyoto | 45.4678 | 11.4397 |
| Li-7 | 39.5586 | 12.9335 |
| Lu3-30 | 30.6814 | 16.2859 |
| Mitterberg-2-184 | 34.3627 | 12.1730 |
| Np-0 | 30.1036 | 13.9691 |
| Obe1-15 | 57.2650 | 14.4833 |
| Pt-0 | 47.6894 | 13.7816 |
| Pu2-8 | 38.6410 | 15.0164 |
| Ru-2 | 42.0965 | 9.8303 |
| Ru4-16 | 42.0428 | 5.7762 |
| Slavi-1 | 15.8131 | 10.2138 |
| Sr:5 | 32.6595 | 10.4755 |
| St-0 | 13.1444 | 3.8517 |
| Ta-0 | 35.6839 | 17.0575 |
| Tu-NK-12 | 39.6717 | 30.2512 |
| UduI 1-11 | 36.2898 | 7.4541 |
| Ull2-3 | 45.5724 | 20.8766 |
| Utrecht | 37.4061 | 3.5961 |
| Wank-2 | 43.6483 | 7.4484 |
| Ws-2 | 38.5694 | 6.7664 |
| ZdrI 1-23 | 38.8363 | 12.4145 |
| ZdrI 2-21 | 24.7347 | 5.7415 |
| AVERAGE | 38.7958 | 12.5604 |
