# Supplementary material for: Parent-of-Origin Effects on Seed Size Modify Heterosis Responses in Arabidopsis thaliana
Source: Front Plant Sci. 2022 Mar 7;13:835219. doi: 10.3389/fpls.2022.835219 (PMC8940307; doi:10.3389/fpls.2022.835219)
Supplement: Supplementary Table 6 — Influence of geolocation on phenotypic variation. Pearson correlation coefficient (r) was used to determine the latitudinal and longitudinal association of the 71 genetically different accessions of this study with reciprocal F1 hybrid diploid seed size, seed size of selfed isogenic diploid parental lines and parent-of-origin effects. Correlation is significant at the 0.05 level. [file Table_6.DOCX]

**Supplementary Table 6.** Influence of geolocation on phenotypic variation. Pearson correlation coefficient (r) was used to determine the latitudinal and longitudinal association of the 71 genetically different accessions of this study with reciprocal F1 hybrid diploid seed size, seed size of selfed isogenic diploid parental lines and parent-of-origin effects. Correlation is significant at the 0.05 level.

|  | **Latitude** | **Longitude** |
| --- | --- | --- |
| **F1 seed size 2x *Ler-0* X 2x Accession** | r = 0.2234 R² = 0.0499 *p* value = 0.0611 | r = 0.1073 R² = 0.0115 *p* value = 0.3732 |
| **F1 seed size 2x Accession X 2x *Ler-0*** | r = 0.1145 R² = 0.0131 p value = 0.3416 | r = -0.0377 R² = 0.0014 *p* value = 0.7552 |
| **Seed size selfed isogenic diploid parents** | r = 0.0909 R² = 0.0083 *p* value = 0.4509 | r = -0.0943 R² = 0.0089 *p* value = 0.4340 |
| **Parent-of-origin effects** | r = -0.337 R² = 0.0011 *p* value = 0.7804 | r = -0.1029 R² = 0.0106 *p* value = 0.3931 |
