# Supplementary material for: Parent-of-Origin Effects on Seed Size Modify Heterosis Responses in Arabidopsis thaliana
Source: Front Plant Sci. 2022 Mar 7;13:835219. doi: 10.3389/fpls.2022.835219 (PMC8940307; doi:10.3389/fpls.2022.835219)
Supplement: Supplementary Table 7 — Broad-sense heritability of hybrid F1 seed size in terms of the parent of origin. [file Table_7.DOCX]

**Supplementary Table 7.** Broad-sense heritability of hybrid F1 seed size in terms of the parent of origin.

| **Cross Direction** | **Broad-Sense Heritability (H^2^)** |
| --- | --- |
| 2x Accession X 2x L*er*-0 | 0.41869 |
| 2x L*er*-0 X 2x Accession | 0.248 |
